# Supplementary material for: Sustained-input switches for transcription factors and microRNAs are central building blocks of eukaryotic gene circuits
Source: Genome Biol. 2013 Aug 23;14(8):R85. doi: 10.1186/gb-2013-14-8-r85 (PMC4054853; doi:10.1186/gb-2013-14-8-r85)
Supplement: Additional file 5 — HTML Browsable Motif Output. Zipped folder containing all WaRSwap and FANMOD motif output, viewable in a web browser. [file gb-2013-14-8-r85-S5.ZIP › HTML_browsable_motif_output/FANMOD_ath_tair9/sigs_fanmodm-2000.pvals.heatmaps.html/motif_id_6_000111002_tftype_ath_upstream_-2000_0.html]

```
BG_MODEL = FANMOD
MOTIF_ID = 6_000111002
TF_TYPE = ath
UPSTREAM = -2000_0


PVals
FN_0.2	FN_0.4	FN_0.6	FN_0.8
dg_60.genes	0.256	0.234	0.141	0.15
dg_70.genes	0.528	0.423	0.262	0.208
dg_80.genes	0	0	0	0

ZScores
FN_0.2	FN_0.4	FN_0.6	FN_0.8
dg_60.genes	0.864	1.124	1.373	1.273
dg_70.genes	0.308	0.756	1.003	0.858
dg_80.genes	2.256	2.207	1.752	1.179

StDevs
FN_0.2	FN_0.4	FN_0.6	FN_0.8
dg_60.genes	385.914	276.042	109.649	35.531
dg_70.genes	239.326	161.666	65.033	20.667
dg_80.genes	5.871	6.05	4.961	2.415
```
